# Empowering Patients and Caregivers to Use Artificial Intelligence and Computer Vision for Wound Monitoring: Nonrandomized, Single-Arm Feasibility Study

**DOI:** 10.2196/69470

**Published:** 2025-06-04

**Authors:** Rose Raizman, José Luis Ramírez-GarciaLuna, Tanmoy Newaz, Sheila C Wang, Gregory K Berry, Ling Yuan Kong, Heba Tallah Mohammed, Robert D J Fraser

**Affiliations:** ^1^Scarborough Health Network, Scarborough, ON, Canada; 2 Lawrence S Bloomberg Faculty of Nursing, University of Toronto, Toronto, ON, Canada; 3 Department of Surgery, McGill University Health Centre, Montreal, QC, Canada; 4 Cumming School of Medicine, University of Calgary, Calgary, AB, Canada; 5 Faculty of Medicine, University of Toronto, Toronto, ON, Canada; 6 Swift Medical Inc, 1 King S W, Suite 4800, Toronto, ON, M4C 4C2, Canada, 1 4168193729; 7 Department of Dermatology, Women's College Hospital, Toronto, ON, Canada; 8 Faculty of Medicine, McGill University, Montreal, QC, Canada; 9 Division of Infectious Diseases, Jewish General Hospital, Montreal, QC, Canada; 10 Arthur Labatt Family School of Nursing, Western University, London, ON, Canada

**Keywords:** artificial intelligence, AI, computer vision, wound care, patient engagement, mobile phone, diabetic foot ulcer

## Abstract

**Background:**

Chronic wounds affect 1%-2% of the global population, and pose significant health and quality-of-life challenges for patients and caregivers. Advances in artificial intelligence (AI) and computer vision (CV) technologies present new opportunities for enhancing wound care, particularly through remote monitoring and patient engagement. A digital wound care solution (DWCS) that facilitates wound tracking using AI was redesigned as a patient-facing mobile app to empower patients and caregivers to actively participate in wound monitoring and management.

**Objective:**

This study aims to evaluate the feasibility, usability, and preliminary clinical outcomes of the Patient Connect app (Swift Medical Inc) in enabling patients and caregivers to remotely capture and share wound data with health care providers.

**Methods:**

A feasibility study was conducted at 2 outpatient clinics in Canada between May 2020 and February 2021. A total of 28 patients with chronic wounds were recruited and trained to use the Patient Connect app for wound imaging and secure data sharing with their care teams. Wound images and data were analyzed using AI models integrated into the app. Clinicians reviewed the data to inform treatment decisions during follow-up visits or remotely. Key metrics included app usage frequency, patient engagement, and wound closure rates.

**Results:**

Participants captured a median of 13 wound images per wound, with images submitted every 8 days on average. The study cohort included patients with diabetic ulcers, venous ulcers, pressure injuries, and postsurgical wounds. A median wound closure surface area closure of 80% (range 15-100) was achieved across all patients, demonstrating the app’s clinical potential. Feedback from patients and clinicians highlighted during the feasibility testing support insight into the app’s usability, data security features, and ability to enhance remote monitoring that need to be explored in further qualitative research.

**Conclusions:**

The Patient Connect app effectively engaged patients and caregivers in chronic wound care, demonstrating feasibility and promising clinical outcomes. By enabling secure, remote wound monitoring through AI technology, the app has the potential to improve patient adherence, enhance care accessibility, and optimize clinical workflows. Future studies should focus on evaluating its scalability, cost-effectiveness, and broader applicability in diverse health care settings.

## Introduction

Chronic wounds are commonly defined as wounds that fail to heal within 4‐12 weeks through normal, timely, and orderly stages [1]. These wounds pose a major public health challenge, with 1%‐2% of the global population estimated to experience a chronic wound during their lifetimes [2]. Diabetic ulcers (DUs), venous ulcers (VUs), and pressure injuries (PIs) are especially prevalent, making up over 90% of all chronic wounds [3] and often require significant wound care management and resources. However, due to their low rate of complete healing, chronic wounds have major impacts on both the health and quality of life of patients and their families, leading to significant issues, such as severe and prolonged pain, loss of function and mobility, amputation, mental health deterioration, social isolation and embarrassment, financial burden, and chronic morbidity or death [4]. Recently, there has been a significant transformation in health care delivery, focusing on remote access through telemedicine that leverages the widespread availability of smartphones and their apps. Technologies that facilitate telemedicine and ensure continuity of care for chronic wound patients are urgently needed, as high risk of wound-related complications exist for those without access to consistent follow-ups [5].

The rise of AI has shown great promise, particularly in the field of wound care. These technologies provide health care professionals with novel tools that contribute towards many improvements in treatment efficiency and efficacy, including early detection, risk factor analysis, prediction, diagnosis, intelligent treatment, outcome prediction, and prognostic evaluation [6]. In addition, AI-powered tools have been shown to empower patients to take control of their own health and well-being. For instance, AI tools can provide patients with information regarding their conditions and treatment options, thereby enabling them to make informed decisions while also strengthening patient-health care provider relationships through trust-building [7]. Computer vision (CV) is a particular form of AI that extracts information from digital images or videos in order to recognize content from visual data [8]. These technologies are especially promising in the field of wound care, as they can help classify wound severity, provide accurate predictions of wound healing, and track changes in wounds over time through image analysis [9,10]. CV technologies have previously been shown to provide significant time savings during wound assessments [11], decrease costs and days needed for wound healing [12], and improve data capture reproducibility and accuracy [13]. Notably, patients have also been found to exhibit positive perceptions toward the use of wound photography in their treatment journeys by helping them track their wound progress or increasing their involvement within their own care [14].

Swift Medical Skin & Wound (hereafter referred to as digital wound care solution [DWCS]) developed a mobile app and dashboard, specifically designed to accurately and reliably measure and document wound characteristics. The system, which is already available and is a privacy-compliant (Health Insurance Portability and Accountability Act and Personal Health Information Protection Act), Health Canada registered and FDA Class I medical device, uses CV technology to automatically focus and calculate wound dimensions from images acquired by the mobile device’s camera, allowing users to obtain precise and consistent measurements. These capabilities have been demonstrated to reduce the time needed to assess the wounds of patients in a more accurate manner [11,15]. In addition, to viewing a wound’s image series over time, additional information such as healing-associated metrics, wound-bed information, anatomical location, and patient identifiers are captured. While the app has provided doctors and wound-care specialists with a powerful assessment solution and a dashboard to remotely monitor and collaborate for an effective wound management strategy, in order to fully realize the system’s potential, patients themselves will need to be able to acquire and securely share images and other relevant information with their care providers. By actively engaging patients in their own wound care journeys through a patient-centric application, individuals may feel empowered to be more active in the treatment process.

**Figure 1.**
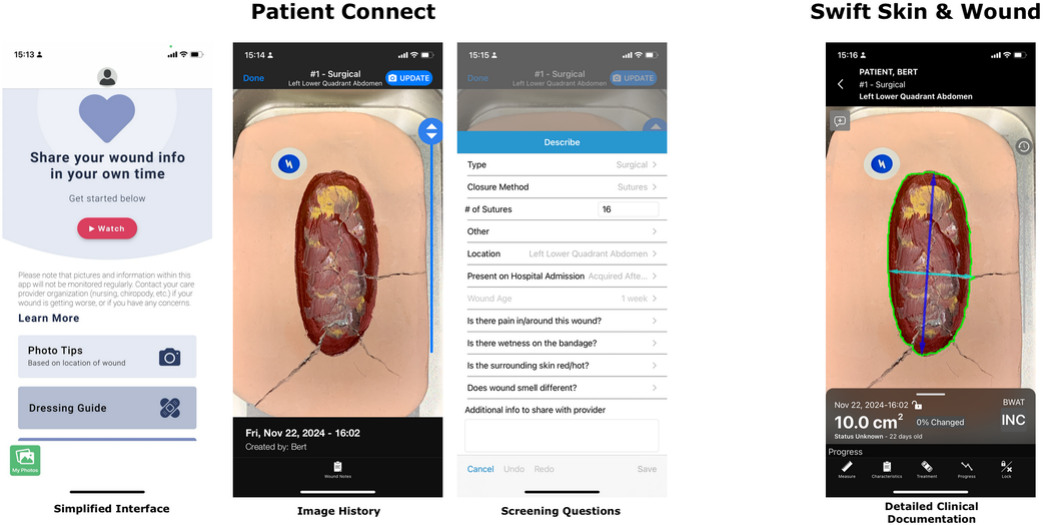
A simulated wound is used to illustrate the difference between the patient-facing mobile app and the clinician mobile apps.

Understanding the importance of innovative technologies in improving health outcomes for chronic wound patients, the DWCS have recently developed a stream-lined, patient-facing version of the AI-powered application called Patient Connect (Swift Medical Inc). Patient Connect is designed for easy use by patients or their care providers using their own personal smartphones, ensuring a more patient-centric approach to wound management (see Figure 1). The user interface (UI) was designed with differences in technology and clinical literary in mind. The DWCS has detailed clinical documentation fields an advanced reporting included. The patient user experience is simplified and provides educational content to support image capture and wound care best practices. The Patient Connect interface had language changes to be grade 3 literacy level accessible. Educational materials including instructional videos and simply language guides for basic wound dressings were included within the app to attempt to improve engagement. The patient image history shows only images and access to information the patient submitted in the documents section, which includes basic screening questions for signs of infection and a free text (see Figure 1; third image from the right). The clinician app has standardized documentation for wound assessment, treatment, and progress to be documented (see Figure 1; first image from the left).

Patients are authorized directly by their health care provider and can only access their own records through their personal device. This requires a 2-step verification via email or a mobile phone number and their date of birth. Like the standard version of the app, it automatically focuses and calculates wound dimensions from the images acquired. Images and other measurements are not stored on the phone camera roll of the patient’s personal devices, instead they are encrypted within the app and securely transmitted to health care providers on the same secure, web-based servers from the DWCS. The patient’s health care provider can access the patient’s generated images and patient-reported data using their app or the web dashboard; thereby, enabling the remote monitoring of wound progression.

The objective of this report is to present results of a feasibility study of early adopters of our patient-centric AI-powered wound assessment technology to image their wound to be included in their medical record and for self-monitoring, within 2 outpatient clinics in a university-affiliated hospital and a community hospital to determine overall feasibility, usability, and preliminary outcomes of the Patient Connect app.

## Methods

### Overview

A nonrandomized, single arm-feasibility study was conducted between May 2020 and February 2021. A nurse practitioner at Scarborough Health Network and 2 physicians at Montreal Jewish General Hospital were the primary clinicians engaged in the project, and both had previous experience using AI-enabled wound care documentation in clinical practice. Standardized training was provided on enrolling patients, enabling access, and reviewing patient-submitted wound images and information in the clinician application and dashboard. Training materials were provided to support patient onboarding to use the service. This included multimedia content (videos on how to download and access the app) that was shared via SMS text messages when the patient was enrolled and content embedded within the app (eg, how to capture wound images). Paper hand out material including instructions were also provided (see sample in Multimedia Appendix 1). Clinicians had access to review images submitted through the dashboard on a weekly basis and during follow-up visits.

A purposive sampling technique was used to recruit patients or caregivers from the Montreal Jewish General Hospital and the Rouge Valley Scarborough Hospital for early testing of the Patient Connect app. A sample size between 20 and 30 participants was determined based on feasibility study design considerations. According to established feasibility study guidelines, sample sizes of 30 or fewer participants may be appropriate for qualitative feasibility studies [16]. This sample size allowed for evaluating the usability, engagement, and feasibility of the intervention while balancing recruitment and resource constraints. Patients were the primary focus for the inclusion criteria, with patient caregivers acting as an inclusion alternative if the patient consented. Inclusion criteria to the cohort were (1) patients’ attending staff were already a user of the DWCS, (2) the patient or a close relative possessed and was familiar with a smartphone device, and (3) the patient had a stable wound, as assessed by their health care provider. Caregivers were considered as an inclusion alternate if the patient consented. Caregivers were suitable alternatives if the wound was in an area that was difficult to image (eg, sacrum and back) or the patient had limitations that made them unable to use the app (eg, mobility and technology literacy). Exclusion criteria were Android phone users as the Patient Connect app currently only runs on iOS devices. In addition, the study excluded patients who did not consent and who did not approve their caregiver to act as an alternate, since, for these patients, caregiver participation was essential for independent app usage. No changes were made to the study methods after the commencement of the study, including eligibility criteria and assessment measurements. All prespecified metrics and inclusion criteria remained unchanged throughout the study period.

**Figure 2.**
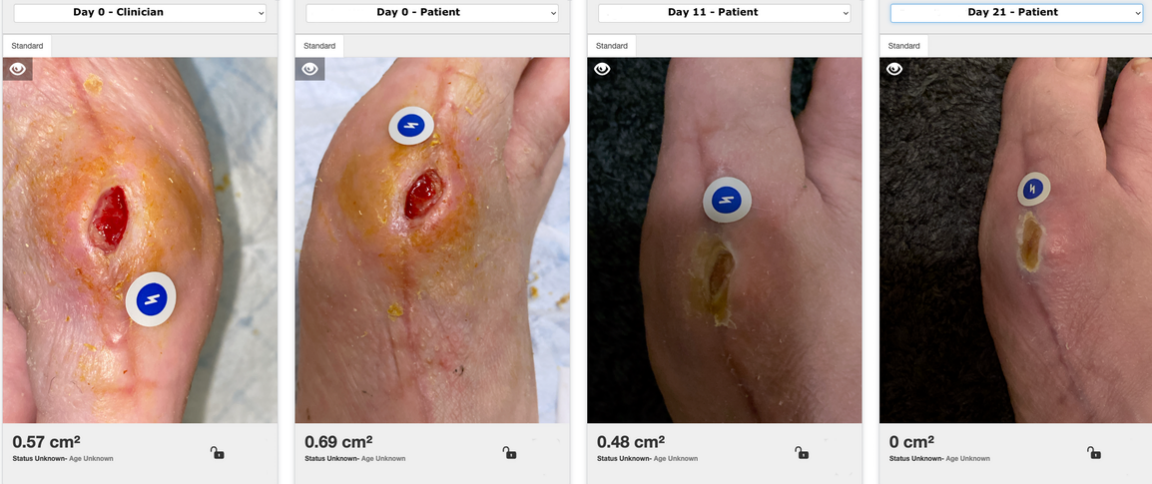
A case series of a postoperative wound. First image on the left was captured by the clinician. Then the patient was taught to capture images and a second image the same day was documented. The 2 images on the right half show follow up monitoring submitted by the patient as the wound closed.

Enrolled participants were encouraged to use the app when their dressing was being changed by themselves, by caregivers, or y bother health care professionals outside of the participating organizations (eg, home health). A 2 case series displaying the measurement and progress tracking of patient-captured and caregiver-captured wound images on the Patient Connect app are shown in Figures2  3, respectively. Due to the variation in wound-changing protocols and the feasibility design, there was no set requirement for imaging completion by the patients per week. However, patients were encouraged to take at least one picture during each wound-changed session. The clinicians collected additional feedback during follow-up appointments. User experience, facilitators, and barriers were documented and shared with the project manager and software development team to support quality improvement and ensure app performance and stability.

Usability metrics were collected to assess feedback on the engagement, consistency, and effectiveness of the tool. These include the frequency app use (ie, the number of wound images uploaded per patient), submission intervals, completion rates of imaging sessions, and tracking adherence rates concerning continued use during the study period. The mean was used to report on continuous or normally distributed variables, and median was used for data with outliers or skewed distribution (eg, wound size and number of images) to minimize influence of extreme values (see Table 1). The app has embedded monitoring software (Mixpanel) for debugging that enabled logging of successful logins, progress through the imaging workflow and deidentified summaries were available to the research team to see counts and frequency of image submission. These features are common practice in mobile and cloud based-software development to identify software issues and iteratively improve user workflows.

In addition, qualitative feedback was collected about ease of use, technical difficulties, general user experience, satisfaction with the tool that was collected during follow-up visits, as well as barriers like light, clarity of images, and comfort level using the app alone. The degree of clinician engagement was assessed by tracking the frequency of image review, using the AI-assisted assessments into treatment decisions, and feedback on patient-submitted data.

The patients were followed until the closure of their wounds or February 2021, whichever occurred first. Wound closure was defined as a wound measurement of 0 cm^2.^ All data included in this report was obtained from the solution’s deidentified servers, allowing for data retrieval while maintaining the confidentiality of patients’ personal information.

**Figure 3.**
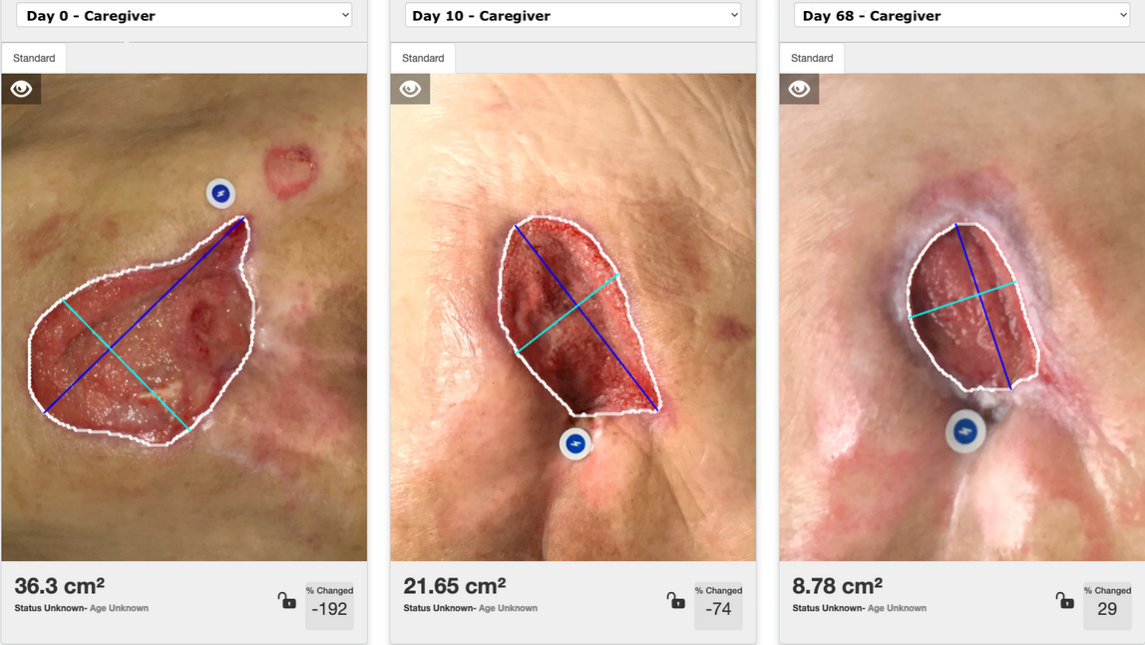
A case series of a hard-to-heal wound on the sacrum imaged by a caregiver during the patient journey. Images have adequate lightening, focus, color correction, and artificial intelligence (AI)-based measurement is shown to the clinician monitoring the wound remotely.

**Table 1.** Patient characteristics. Data are presented as mean (SD), median (range), or proportions.

Variable	Results (N=28)
Age (years), mean (SD)	66.4 (18.5)
Gender, n(%)	
Female	14 (52)
Male	13 (48)
Type of lesion, n (%)	
Diabetic ulcer	14 (52)
Venous ulcer	7 (26)
Pressure ulcer	4 (15)
Postsurgical	2 (7)
Initial wound size (cm^2^), median (range)	3.71 (0.48-27.91)
Follow up time (months), median (range)	3 (1-9)
Number of images submitted, median (range)	13 (4-45)
Average time between images (days), median (range)	8 (3-14)
Percentage of wound closure achieved (%), median (range)	80 (15-100)

### Ethical Considerations

The study received multisite ethics approval provided by the Scarborough Health Network Research Ethics Board (SUR-21‐007). Patient or substitute decision-makers provided consent and had the ability to withdraw at any time. Data from subjects that withdrew would be excluded from analysis and their data would not be used for secondary analysis without their consent.

## Results

### Patient Characteristics

A total of 28 patients adopted the Patient Connect App as early users. The cohort included patients with varied wound types, including diabetic foot ulcer (DFU), venous leg ulcer (VLU), PI, and surgical wounds. The characteristics of the wounds are presented on Table 1.

Approximately half of the patients were diabetics with plantar ulcers (52%, n=14). There was a balanced gender mix in this study, with 52% (n=14) of patients reporting as males and 48% (n=13) as females. The sample population had a range of wound sizes from 0.48 cm^2^ to 27.91 cm^2^ and a median size of 3.71cm^2^ (6.17 cm^2^). Wound measurement was captured from photographs using AI models, so wounds outside of the photograph (ie, circumferential) had limitations to their data. This suggested that single-surface wounds were optimal for patient and caregiver imaging and automated AI analysis of the wound. Wound imaging was found to be ideally suited for patients with images on a single surface. However, it was possible to upload multiple images if wounds were circumferential.

The median follow-up was 3 months, with a median of 13 images aquired by the patient or caregiver per wound. Images were captured on average every 8 days. Interestingly, despite a general infrequency of in-person follow-up visits, the median wound closure rate recorded in the app was 80% (IQR 15%‐100%). No adverse events or unintended harms were reported among participants.

### Projected Cost Savings

The Patient Connect app enables remote monitoring of the wounds and reduces the need for in-person visits and related costs. With patients documenting a median of 13 images per wound over 3 months, this assessment could replace several visits to the clinic. Assuming that each time a picture is submitted, 1 trip is saved, that could mean there is the possibility of eliminating up to 13 trips per patient, representing savings anywhere between US $140 and US $281 in travel costs per patient (with an average travel cost of US $10.82 per visit) [17,18]. As for the sample of this study consisting of 28 patients, this would mean US $3931 to US $7862 in total travel savings over the three months. Savings could amount to US $140,000-$281,000 with 1000 users in a year.

In addition, fewer trips would equate to fewer hours lost at work for both patients and caregivers. Assuming 2 hours off work per visit at an average hourly wage of $36.64 CAD , with 13 visits avoided, a direct saving of $595 per missed trip or $16,674 could be achieved for the study cohort. A scale of 1000 users would mean savings of $595,000/year in workforce productivity.

### User Experience and Quality Improvement Insights

Patient feedback on Patient Connect was useful in determining usability, engagement in wound care, and areas for improvement. Many participants noted that remote wound image capture and sharing opened their eyes to changes in the wound that made them more active in the wound care process and compliant with treatment. Some patients reported that taking pictures regularly helped monitor their healing and increase their motivation to adhere to wound care protocols such as the frequency of dressing changes, hygiene practices, and alleviating pressure techniques.

Although Patient Connect appeared useful in many aspects, several issues came to light. Literacy and accessibility problems were felt, particularly among older adults or other patients unfamiliar with smartphone apps, who sometimes required caregiver assistance to capture and submit images of their wounds. Patients had difficulty taking clear pictures if the wounds were in hard-to-reach areas (eg, sacrum, back, or heels) and tended to submit images erratically. Lighting posed challenges since some patients had difficulty ensuring adequate exposure for accurate AI analysis. While many people found the app helpful, some users experienced fatigue with engagement and became less consistent in taking images, especially if slow healing of the wound was involved. A few participants expressed common data privacy concerns about sharing images digitally, while continued education on encryption and security protocols was offered to help provide reassurance.

## Discussion

### Principal Findings

In this report, we demonstrate that the Patient Connect’s regular use by a group of selected patients allowed the remote monitoring of their wounds, successfully capturing medical-grade images that were subsequently used by clinicians for treatment decisions. This capability is not only crucial for maintaining continuity of care but also for enhancing patient engagement and treatment adherence, as evidenced by the increase in image sharing and self-monitoring behavior. The app facilitated the collection and analysis of data, which was instrumental in improving patient behavior and health outcomes by providing real-time feedback and enabling timely communication through wound status updates with health care professionals.

Patients using the Patient Connect app exhibited a high frequency of engagement with the AI software, submitting an average of 13 pictures, or 1 image every 8 days to clinicians throughout the duration of their wound care. In addition, a median wound closure rate of 80% (IQR 15-100) was observed across all patients and wound types. These findings suggest that the use of the Patient Connect app for participants may have supported engagement with monitoring wound healing, which may have influenced better healing outcomes across the diverse wound types. It is recognized, however, that factors such as standard wound care practices, clinical interventions, and individual patient conditions may have influenced the results. Clinical decisions within wound care may be delayed without adequate history. Patients in the study enabled a better record of the wound’s response or lack of response to treatment that may support more timeline adjustments in care, which could be better understood through future research.

Interestingly, our results align with findings from other smartphone-based AI treatment platforms. For instance, Labovitz et al [19] demonstrated that, among patients with recently diagnosed ischemic strokes receiving anticoagulants, real-time monitoring via a smartphone-based AI app led to significantly improved medication adherence. This intervention resulted in a 50% increase in adherence rates compared to the standard care control group, as measured by plasma drug concentration levels.

Our findings also align with previously published results demonstrating the potential of the patient-centered digital wound care technology for remote wound monitoring. For example, a case study by Kong et al [20] highlighted the successful application of the DWCS technology in the management of a male patient with type 1 diabetes and multiple comorbidities, including chronic kidney disease and a previous toe amputation. Initially managed for osteomyelitis of a chronic foot ulcer via text and email, the patient transitioned to using the DWCS Patient Connect app for monitoring and management between June 2020 and January 2021. Over 7 months, the patient submitted 39 wound images—a nearly 20-fold increase in the sharing of wound-related data compared with the situation before using the app—enabling the tracking of accurate measurements of 2 additional wounds. The app fostered patient engagement through weekly assessments, promoting self-examination, and preventive behaviors such as infection and trauma monitoring and off-loading of wound pressure through orthotics. Remote follow-ups reduced health care visits, alleviating patient anxiety by minimizing direct contact and enhancing physicians’ confidence to deliver effective care remotely. Streamlined workflows and the use of images captured during dressing changes further saved time and costs, demonstrating the app’s potential to optimize wound management and expand care capacity. The patient also found the app “educational and empowering,” highlighting the ability of patient-centred technology to improve patient sentiment and better engage individuals with their wound care treatments.

In Kong and colleagues’ case study [20], the assessed patient expressed concerns about sharing wound images via standard messaging platforms, highlighting a common issue with smartphone-based remote care strategies: the security of patient data [21]. Before transitioning to the app, the patient, despite having direct access to their physician, felt that sending images could impose on the physician’s time. In addition, the patient was uncomfortable with the idea that the images would be transmitted through standard messaging and stored on the physician’s smartphone, raising privacy and data security concerns. In contrast, by storing images captured using the app on secure cloud-based servers, this reduced the patient’s anxiety toward sharing images and facilitated the physician’s ability to rapidly and securely receive images.

While the sample size is small, this pilot study provides promising results regarding the use of the Patient Connect app. Our findings demonstrate that the app can be effectively used across various types of wounds and health care settings. It has been used in hospital departments, such as the Division of Infectious Diseases at the Jewish General Hospital, as well as in ambulatory settings, including ostomy care and pressure ulcer prevention at Centenary Hospital, Scarborough Health Network, and Ontario Health at Home. No adverse outcomes or wound complications were recorded with the use of the Patient Connect app during the study period. No significant privacy or security issues arose as well as the app followed all regulatory protocols regarding data protection. However, a few participants, usually elderly patients, may have highlighted the need to use assistance in taking pictures of wounds for difficult to reach or seen areas such as the sacrum or back. Lighting conditions also had an effect on the quality of the images, which indicated the need for further instruction or caregiver assistance in cases where optimal image capture was crucial.

Future studies are needed to rigorously evaluate the time savings associated with the use of the app, such as reductions in days lost due to unplanned hospital admissions or the average number of missed workdays. In addition, research should investigate whether incorporating the app as part of a remote wound care strategy can deliver care that is comparable to or even superior to standard in-person appointments by measuring median days to heal and wound complication rates. Beyond clinical outcomes, the app’s potential to reduce patient costs related to travel, time off work, and other logistical burdens associated with frequent health care visits highlights its value in remote care settings. As this study had a 3-month follow-up period, which may not fully capture the healing trajectory or wound recurrence for some wound types, an extended follow-up duration is recommended in future studies. Such insights will be critical in validating the app’s role in enhancing accessibility, efficiency, and cost-effectiveness in wound care. In addition, we are currently exploring the potential use cases of our technology for postsurgical sites, aiming to evaluate the effectiveness and feasibility of patient-centered wound images to detect infection. Understanding the potential use cases of generative AI for patient support may also be a worthwhile avenue for further exploration, for example, summarizing the AI analysis of the images captured by patients and providing information on the next steps (eg, clinician follow-up or continued self-management). AI and CV technology may offer patients and caregivers meaningful tools that empower them to understand better their condition, treatment options, and progress addressing gaps that chronic wounds face due to falling outside of a medical specialty. Furthermore, this study explained and discussed the development of the Patient Connect app for feasible remote wound monitoring. Swift Medical further introduced advanced AI-enhanced features such as AutoDepth and SmartTissue to deal with any challenges surrounding the monitoring of complex wounds. For example, AutoDepth identifies wound edges, calculates dimensions, and pinpoints the deepest area of the wound in real-time. SmartTissue is capable of quantifying tissue types, namely, epithelial, granulation, slough, and eschar—irrespective of the skin tone (Gupta et al [22]). These innovations enhance precision, introduce automation, and facilitate clinical decision-making. Future studies should examine the effect of the innovations on patient engagement, complex wound assessment, and treatment outcomes.

### Limitations

This study was limited to a targeted patient group of 28 individuals across two hospitals, which may restrict the generalizability of our findings. In addition, while images were collected from a variety of wound types, further research is needed to evaluate the applicability of the technology for complex versus simple wounds and location of wounds. For example, situations may exist where caregiver support would be necessary like for wounds in inaccessible locations. However, differences in patient and caregiver technical proficiency with smartphones and apps were not standardized or controlled for as potential confounding factors. Furthermore, understanding the relationship between the technological capability and the app’s use, engagement level, and clinical outcome would provide valuable insight. Future studies could help inform the creation of training programs to increase adoption and usability in various patient and caregiver populations. In addition, the study only included patients using iOS devices, potentially excluding the experience from a broader population who use Android or other platforms. Future research should evaluate the feasibility and usability, as well as the clinical advantages, of an Android-compatible version. Furthermore, cross-platform studies comparing user experiences and engagement between iOS and Android users might give insight into possible differences in adoption, functionality, and effectiveness for remote wound monitoring.

Due to the nature of this as a feasibility study, the absence of a control group limits the ability to infer causality from the Patient Connect app to wound healing outcomes. However, feasibility studies are still important as they inform and guide the design of future large-scale trials. The findings from this study, where an observed median wound closure rate was 80% (IQR 15%-100%), offer preliminary insights into potential benefits. Such data could facilitate a sample size estimation in a randomized controlled trial to be run in the future. Sample size calculation suggests that 81 per group (162 total) would be required to have a power of 80% to detect a statistically significant difference between wound healing outcomes in the intervention and standard care without it done with a level of significance of 5% (α=.05), assuming a healing rate of 60% with standard care without intervention. These findings should be further investigated to understand their validity, as well as some other broader clinical and economic implications.

### Conclusion

AI-powered medical tools exhibit tremendous potential in their ability to promote treatment optimization, patient satisfaction, treatment adherence, and overall health outcomes. Our pilot study found numerous clinical benefits using the novel patient-centered, CV-powered mobile app for chronic wound assessment. Similarly, the regular image capture by patients enabled physicians to conduct real-time wound assessments, thereby increasing patient adherence to management plans, as evidenced by an 80% wound closure rate within the participating sample. Considering the potential for technologies like the Patient Connect app to positively impact patient behavior and involvement within their own health care treatment journeys by collecting data that benefits their own self-awareness and clinical decision-making, future research should be conducted to understand the clinical, operational, and financial outcomes impacted by patient self-monitoring of wounds and chronic wounds. Factors that would help the widespread adoption of this innovation include more evidence-based research from larger patient populations to demonstrate the app’s effectiveness and benefits in helping deliver remote care, continued user-interface improvements, further maturation of the AI wound assessment technology, patient education on the use of apps and general improvements in specific populations (eg, the elderly) familiarity with technology, and access to high-speed internet, especially for rural populations.

## Supplementary material

10.2196/69470 Multimedia Appendix 1 Patient Connect instructions for patient or care giver support in adoption of AI-powered wound self-monitoring solution.

## Abbreviations

AI: artificial intelligence
CV: computer vision
DFU: diabetic foot ulcer
DU: diabetic ulcer
DWCS: digital wound care solution
PI: pressure injury
UI: user interface
VLU: venous leg ulcer
VU: venous ulcer
