# Supplementary material for: Empowering Patients and Caregivers to Use Artificial Intelligence and Computer Vision for Wound Monitoring: Nonrandomized, Single-Arm Feasibility Study
Source: J Particip Med. 2025 Jun 4;17:e69470. doi: 10.2196/69470 (PMC12157955; doi:10.2196/69470)
Supplement: Multimedia Appendix 1 [file jopm-v17-e69470-s001.pdf]

SWIFT SKIN AND WOUND

Using the Patient Connect App

Share important indicators of your wound's healing with your provider.

STEP 1

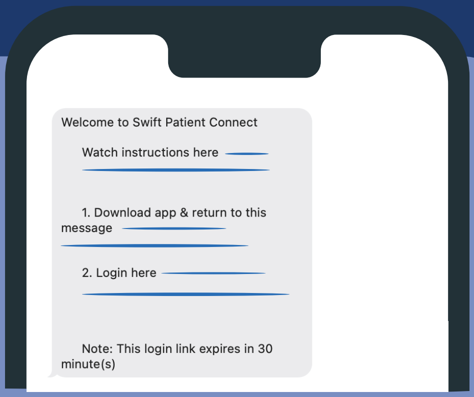

Install the Swift Patient Connect app on your iPhone or iPad device. Your wound care provider will ensure that you receive an email and a text message to your smartphone. The e-mail and text message will contain instructions and login links. Tap the first link to download the app onto your device. Once the app is downloaded, tap the login link. You'll be prompted to verify your date of birth and to accept the Terms of Service. You're now ready to start using the Swift Patient Connect app!

For future logins, the app will require you to enter an additional unique 6-digit code. This code will be sent via text message and e-mail each time you try to login.

STEP 2

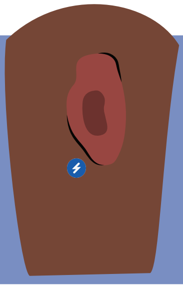

Place your HealX marker next to the edge of your wound on healthy skin. This marker is important for automatically providing the true colour and size of the wound to your provider. Remember to use a new HealX marker each time.

STEP 3

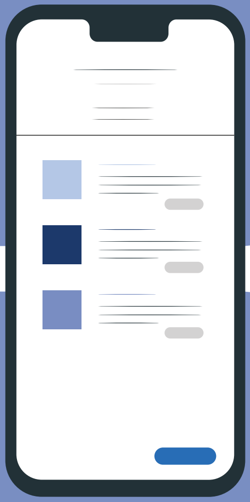

Log in to the Swift Patient Connect app and tap on the green **My Photos** button. You will see that your provider has already captured photos of your wound. Your goal is to continue the wound photo timeline.

STEP 4

Use the photo and location name to confirm that you are updating the correct wound. Tap **Update Wound** to be taken to the camera view.

If you'd like to take a photo of a new wound instead, tap the **New Wound** button in the right-hand bottom corner.

STEP 5

Hold the phone parallel to the wound. Make sure the entire wound is in the view and the HealX marker is highlighted green. To capture a photo, tap the shutter button or press anywhere on the screen for 3 seconds. The app will automatically take a photo. If you're not happy with your photo, you can tap the **Discard & Retake** button. Otherwise, tap **Save & Share**.

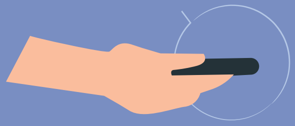

STEP 6

You'll be prompted to fill out some questions about your wound. The only required question is "**is there pain in/around this wound?**". When you tap on that question, you can answer **Yes** or **No**. If you tap Yes, you can describe more about that pain.

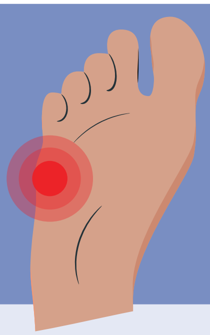

STEP 7

Tap **Done**. Tap **Save**. You'll be taken to the wound timeline view. All of the information you just collected will be directly sent to your provider. If you would like, you can use the scroller to scroll down to view previous photos. Once you're done, tap **Done** to return back to your photos page.

It's important to note that the Patient Connect doesn't show any wound measurements in the app. This information will be relayed to your provider privately.

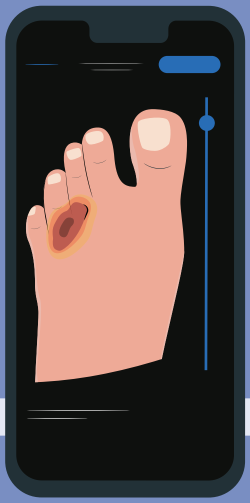

QUESTIONS?

Check out the Swift Patient Connect FAQ section. We also encourage you to watch our instructional video, which can be accessed on the app's homepage via the **Watch** button. If you can't find your answer, contact your wound care provider to learn more.

For technical support, please contact: connect@swiftmedical.com
